# Triclabendazole disrupts mitochondrial electron transport *in vitro*

**DOI:** 10.1128/aac.01756-25

**Published:** 2026-04-30

**Authors:** Tobias Kämpfer, María Eugenia Ancarola, Pascal Zumstein, Alice Bernal, Trix Zumkehr, Seraina Mühlemann, Natalie Wiedemar, Britta Lundström-Stadelmann

**Affiliations:** 1 Department of Infectious Diseases and Pathobiology, Vetsuisse Faculty, Institute of Parasitology, University of Bern 685610, Bern, Switzerland; 2 Graduate School for Cellular and Biomedical Sciences (GCB), University of Bern 27210, Bern, Switzerland; 3 Multidisciplinary Center for Infectious Diseases, University of Bern 27210, Bern, Switzerland; The Children's Hospital of Philadelphia, Philadelphia, Pennsylvania, USA

**Keywords:** succinate dehydrogenase, triclabendazole, parasite, helminth, anthelmintic, complex II, benzimidazole, *Echinococcus*

## Abstract

Mitochondrial respiration, which relies on succinate dehydrogenase (SDH), among other factors, is essential for cellular energy metabolism. This study shows that triclabendazole and its primary metabolites inhibit SDH in mitochondria of the cestode *Echinococcus multilocularis*, while other benzimidazoles do not. Similar effects were observed in mitochondria from additional helminths (trematodes, nematodes) and mammalian cells. These findings indicate a shared mode of action and highlight potential safety risks associated with long-term triclabendazole treatment in mammals.

## INTRODUCTION

In eukaryotic organisms, energy generation primarily relies on mitochondrial oxidative phosphorylation, which integrates the electron transport chain (ETC) and substrate-level phosphorylation. Electrons pass through enzyme complexes (I–IV) to the final acceptor, oxygen. This creates a proton gradient across the inner mitochondrial membrane that powers ATP-synthase (complex V) (1). As many parasites depend on mitochondrial energy metabolism during specific life cycle stages, and their mitochondrial components often differ from those of mammalian hosts, the ETC has emerged as a selective drug target: several antiparasitic agents (2–5) act on complex III (cytochrome *bc*_1_), leading to rapid energy depletion and parasite death. Another promising target is mitochondrial complex II, which acts as a succinate dehydrogenase (SDH). By oxidizing succinate to fumarate, it links the citric acid cycle with oxidative phosphorylation. In helminths and other organisms living under low-oxygen conditions, this enzyme can catalyze the reverse reaction, acting as a fumarate reductase with fumarate as the terminal electron acceptor (6). It is essential for the energy metabolism of many helminths and represents an attractive, potentially druggable target (7, 8).

*Echinococcus multilocularis* causes alveolar echinococcosis, a severe zoonosis with infiltrative, tumor-like growth of larval cysts (metacestodes) in the liver that leads to a fatal outcome if untreated (9). Drug treatment options are limited to the benzimidazoles (BMZs) albendazole and mebendazole, which are parasitostatic, often cause side effects, and rarely achieve cure (10), highlighting the need for safer and more effective drugs. BMZs inhibit microtubule polymerization (11), but may also target other pathways in helminths (12), including mitochondrial complex II (13–15). To explore this, we screened 20 BMZs (Table 1) for activity on SDH in mitochondria from *E. multilocularis* metacestode vesicles, other helminths (trematodes and nematodes), and mammalian cells.

**TABLE 1:** List of tested benzimidazoles

Benzimidazole	Abbreviation	Supplier
Albendazole	ABZ	Merck, Buchs, Switzerland
Ricobendazole	ABZ-SO	Lucerna-Chem, Lucerne, Switzerland
Albendazole sulfone	ABZ-SO_2_	Lucerna-Chem, Lucerne, Switzerland
Benomyl	BNM	Lucerna-Chem, Lucerne, Switzerland
Cambendazole	CMB	Lucerna-Chem, Lucerne, Switzerland
Carbendazim	CZM	Lucerna-Chem, Lucerne, Switzerland
Fenbendazole	FBZ	Merck, Buchs, Switzerland
Febantel	FBT	Merck, Buchs, Switzerland
Mebendazole	MBZ	Lucerna-Chem, Lucerne, Switzerland
Nocodazole	NCZ	Lucerna-Chem, Lucerne, Switzerland
Oxibendazole	OBZ	Lucerna-Chem, Lucerne, Switzerland
Oxfendazole	OFZ	Merck, Buchs, Switzerland
Omeprazole	OMP	Lucerna-Chem, Lucerne, Switzerland
Parbendazole	PBZ	Lucerna-Chem, Lucerne, Switzerland
Triclabendazole	TCBZ	Lucerna-Chem, Lucerne, Switzerland
Triclabendazole sulfoxide	TCBZ-SO	Merck, Buchs, Switzerland
Triclabendazole sulfone	TCBZ-SO_2_	Merck, Buchs, Switzerland
Thiabendazole	THB	Lucerna-Chem, Lucerne, Switzerland
Flubendazole	UBZ	Lucerna-Chem, Lucerne, Switzerland

*E. multilocularis* metacestode vesicles were cultured according to Spiliotis et al. (16). Adult *Fasciola hepatica* flukes isolated from the bile ducts of naturally infected cattle livers (obtained from abattoir Bell, Oensingen, Switzerland) and adult *Toxocara cati* (from the diagnostic unit of the Institute of Parasitology, University of Bern, Switzerland) served as trematode and nematode models, respectively. Mammalian controls included mitochondria from rat Reuber hepatoma H-4-II-E cells (RH cells, *Rattus norvegicus*, ATCC, LGC Standards S.a.r.l., Molsheim Cedex, France) and liver tissue from healthy female mice (*Mus musculus*, CD-1 IGS strain, Charles River Laboratories, Germany).

SDH activity was measured fluorometrically via irreversible reduction of resazurin to resorufin (17), preventing reverse complex II activity. As a control, an assay with triclabendazole (TCBZ), no mitochondria, and alternative reducing agents was performed (Fig. S1). Mitochondria were prepared by tissue homogenization in buffer (Tris-HCl 15 mM, pH 7.4, sucrose 0.33 M, EDTA 25 µM) with a glass-Teflon homogenizer, followed by centrifugation at 1,000 ×*g* for 10 min at 4°C. Enriched mitochondrial fractions were diluted to 10 mg/mL in hypotonic buffer (Tris-HCl 10 mM, pH 7.5) and used at 0.1 mg/mL in 100 µL assay buffer (50 mM potassium phosphate pH 7.5, 1 mM MgCl_2_, 5 mM NaN_3_, 27.5 mM resazurin) with either 2% DMSO (negative control), 2% DMSO + 100 mM sodium malonate (positive control), or 2% DMSO + 40 µM BMZ. Reactions were started with 40 mM succinate and measured in 96-well plates with three biological replicates and technical triplicates. Fluorescence (*λ*_ex_ = 544 nm, *λ*_em_ = 590 nm) was measured every 30 s for 5 min before and for 10 min after initiation (Hidex Sense Reader, Hidex Oy, Turku, Finland). Relative SDH activity was expressed as fluorescence change after succinate addition relative to the negative control. Considering that previous studies have demonstrated interference of uncouplers with resazurin-based assays (18), we acknowledge that this assay may not discriminate between specific effects on SDH and general mitochondrial uncoupling.

**Fig 1:**
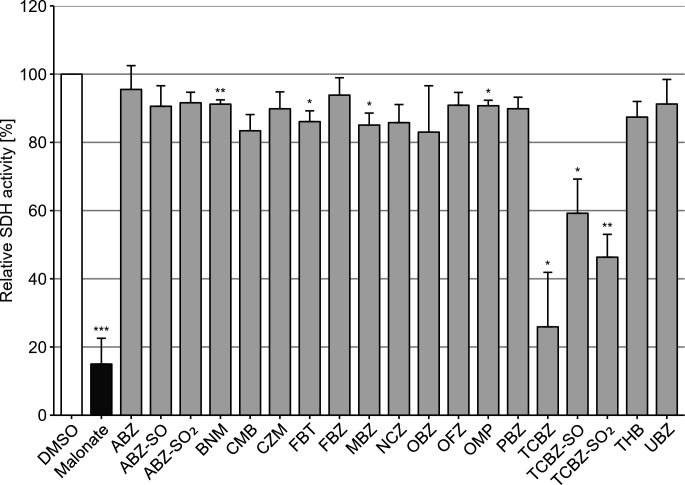
SDH activity assays performed on enriched mitochondrial fractions from *E. multilocularis* metacestode vesicles. Measurements were performed in biological and technical triplicates. Data are shown as mean fluorescence increase relative to the negative control + SD. Results with Bonferroni-adjusted *P*-values below 0.05 (*), 0.01 (**), and 0.001 (***) were regarded as significant.

The BMZs were screened on mitochondria enriched from *E. multilocularis* metacestode vesicles (Fig. 1). The competitive complex II inhibitor (malonate), as well as TCBZ and its metabolites (TCBZ-SO and TCBZ-SO_2_) significantly inhibited SDH activity compared to the DMSO negative control (malonate: 15.0%; TCBZ: 25.9%; TCBZ-SO: 59.2%; TCBZ-SO_2_: 46.3%; see Fig. S2 for data and respective *P*-values), and this response was dose dependent (Fig. S3). In contrast, all other BMZs showed SDH activity above 80%. A structure–activity relationship analysis was beyond the scope of this study and would require a substantially broader data set. However, the presence of the methylmercapto group in TCBZ represents a key structural distinction from the other BMZs tested here and may contribute to its differing activity toward SDH. Richter et al. previously reported on the *in vitro* activity of TCBZ against *E. multilocularis* metacestodes vesicles, with effective concentrations comparable to those applied in the present study (19). These values are likewise consistent with concentration ranges reported for BMZs in earlier *in vitro* investigations (20).

**Fig 2:**
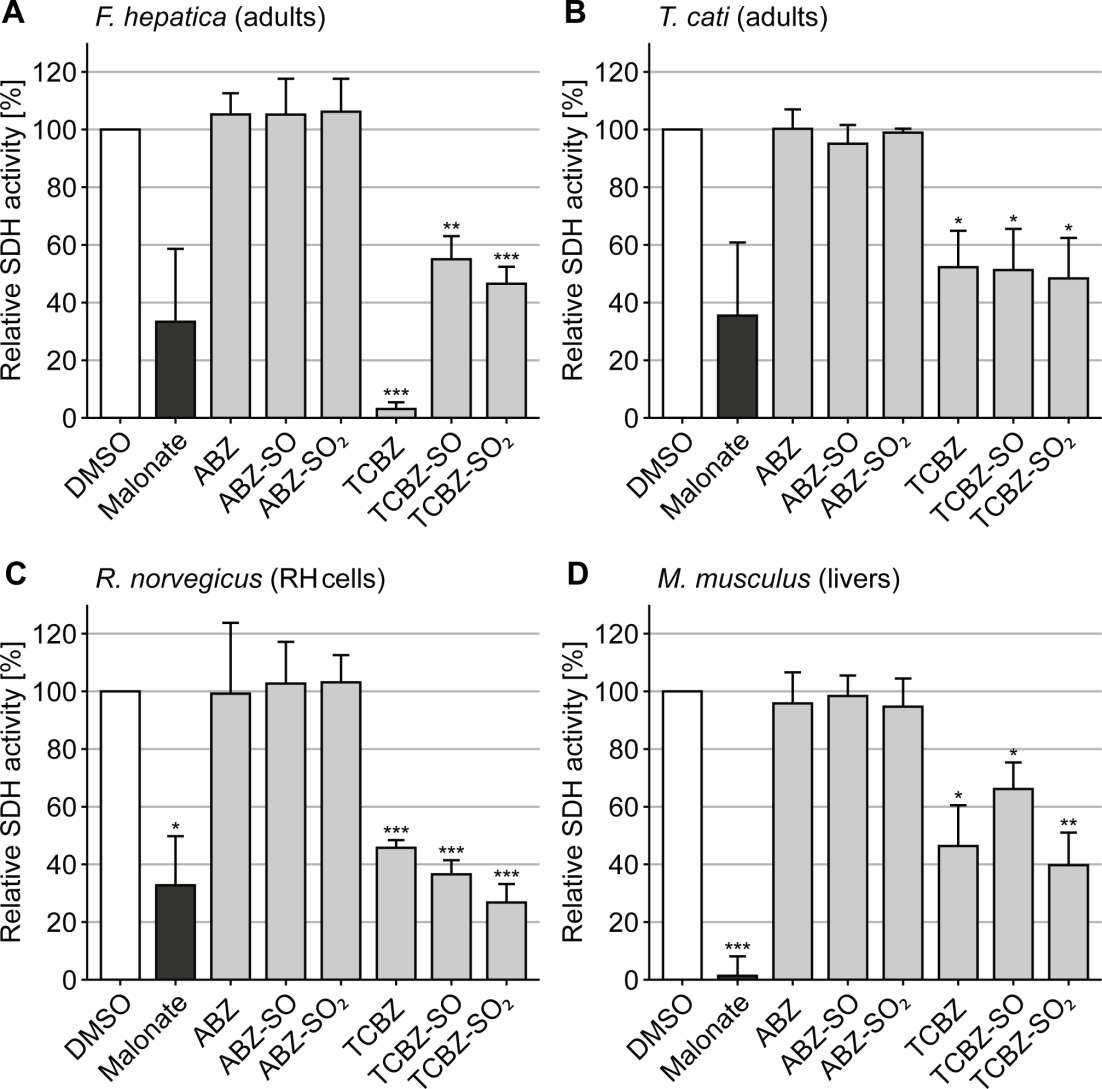
SDH activity assay performed on enriched mitochondrial fractions from adult flukes of *F. hepatica* (**A**), adult worms of *T. cati* (**B**), cultured hepatoma cells of *R. norvegicus* (**C**), and livers of mice (*M. musculus*) (**D**). Measurements were performed in biological and technical triplicates. Data are shown as mean fluorescence increase relative to the negative control + SD. Results with Bonferroni-adjusted *P*-values below 0.05 (*), 0.01 (**), and 0.001 (***) were regarded as significant.

To further explore this observation, we tested TCBZ, albendazole (ABZ), and their respective metabolites on mitochondria-enriched samples from other organisms (Fig. 2). TCBZ, its primary metabolites, and the malonate control consistently inhibited SDH activity across all tested helminth species and mammalian models. Significant inhibition was observed in *F. hepatica* (TCBZ: 3.08%; TCBZ-SO: 55.0%; TCBZ-SO_2_: 46.6%), *T. cati* (TCBZ: 52.3%; TCBZ-SO: 51.3%; TCBZ-SO_2_: 48.4%), *R. norvegicus* (TCBZ: 45.8%; TCBZ-SO: 36.6%; TCBZ-SO_2_: 26.8%), and *M. musculus* (TCBZ: 46.4%; TCBZ-SO: 66.1%; TCBZ-SO_2_: 39.7%; see Fig. S2 for data and respective *P*-values). In contrast, ABZ and its metabolites showed no significant effect on SDH activity.

These findings show that TCBZ targets complex II in a broad, species-independent manner across all tested helminth groups and also in mammals. This contrasts with earlier studies, which primarily focused on nematodes (14, 21–25). However, it has to be acknowledged that the lack of full dose-response curves limits a quantitative comparison of relative potency. Moreover, TCBZ has been reported to act as an uncoupler, and as aforementioned, the assay applied here cannot exclude the possibility that such activity also affected SDH function (26).

**Fig 3:**
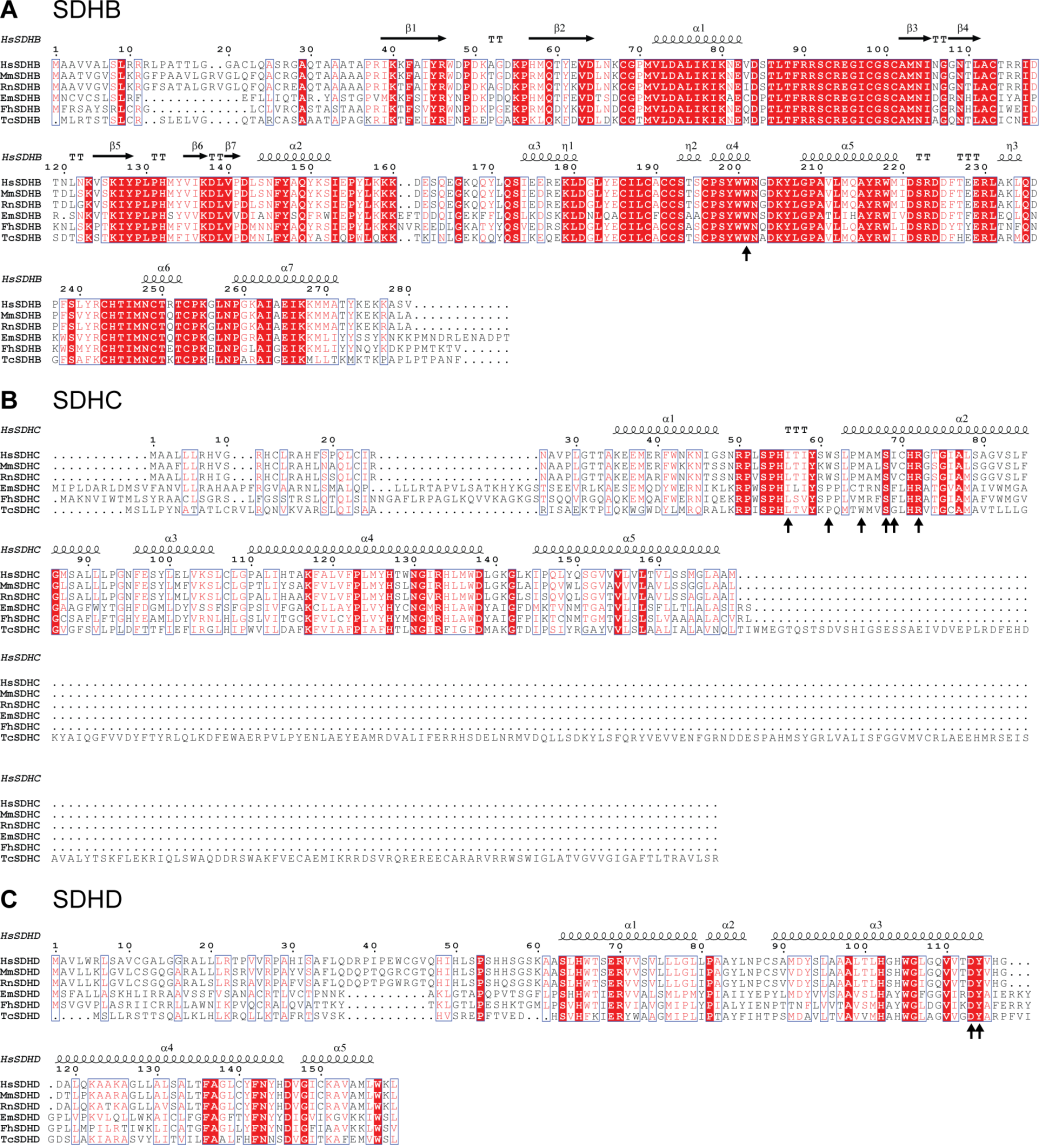
Multiple sequence alignment of SDH subunits (**A**, SDHB: succinate dehydrogenase [quinone] iron-sulfur subunit;** B**, SDHC: succinate dehydrogenase cytochrome b560 subunit; **C**, SDHD: succinate dehydrogenase [quinone] cytochrome b small subunit). Red indicates identical residues, while red letters indicate similar residues. Black arrows indicate the quinone-binding site according to Inaoka et al. (29). As *T. cati* sequences were unavailable, *T. canis* sequences were used instead.

To assess potential structural explanations, we compared SDH subunit sequences using BioEdit (v7.2), with visualization in ESPript 3.0 (Fig. 3). Sequences were retrieved from UniProt (Fig. S4), with secondary structure information from the human complex II (PDB:8GS8). Attention was given to subunits B–D, which form the quinone-binding site, a described target of thiabendazole (13, 27). Alignments for subunit A are shown in Fig. S5. Most subunits were conserved, but distinct residue changes were observed in the quinone-binding site of helminths compared with mammals, consistent with their use of alternative quinones, such as rhodoquinone (8, 28).

TCBZ is currently used to treat *F. hepatica* infections usually as a well-tolerated single dose (30). In contrast, treatment of tissue-dwelling parasites like *E. multilocularis* requires prolonged, multi-dosage regimens with other BMZs (10). Based on our findings, we advise caution regarding the long-term application of TCBZ in mammalian hosts, as toxic effects on mitochondrial function may occur.

## SUPPLEMENTAL MATERIAL

Supplemental material aac.01756-25-s0001.pdf Supplemental files S1 to S5.

ASM does not own the copyrights to Supplemental Material that may be linked to, or accessed through, an article. The authors have granted ASM a non-exclusive, world-wide license to publish the Supplemental Material files. Please contact the corresponding author directly for reuse.
