# Supplementary material for: Triclabendazole disrupts mitochondrial electron transport in vitro
Source: Antimicrob Agents Chemother. 2026 Apr 30;70(6):e01756-25. doi: 10.1128/aac.01756-25 (PMC13231881; doi:10.1128/aac.01756-25)
Supplement: Supplemental material — Supplemental files S1 to S5. [file aac.01756-25-s0001.pdf]

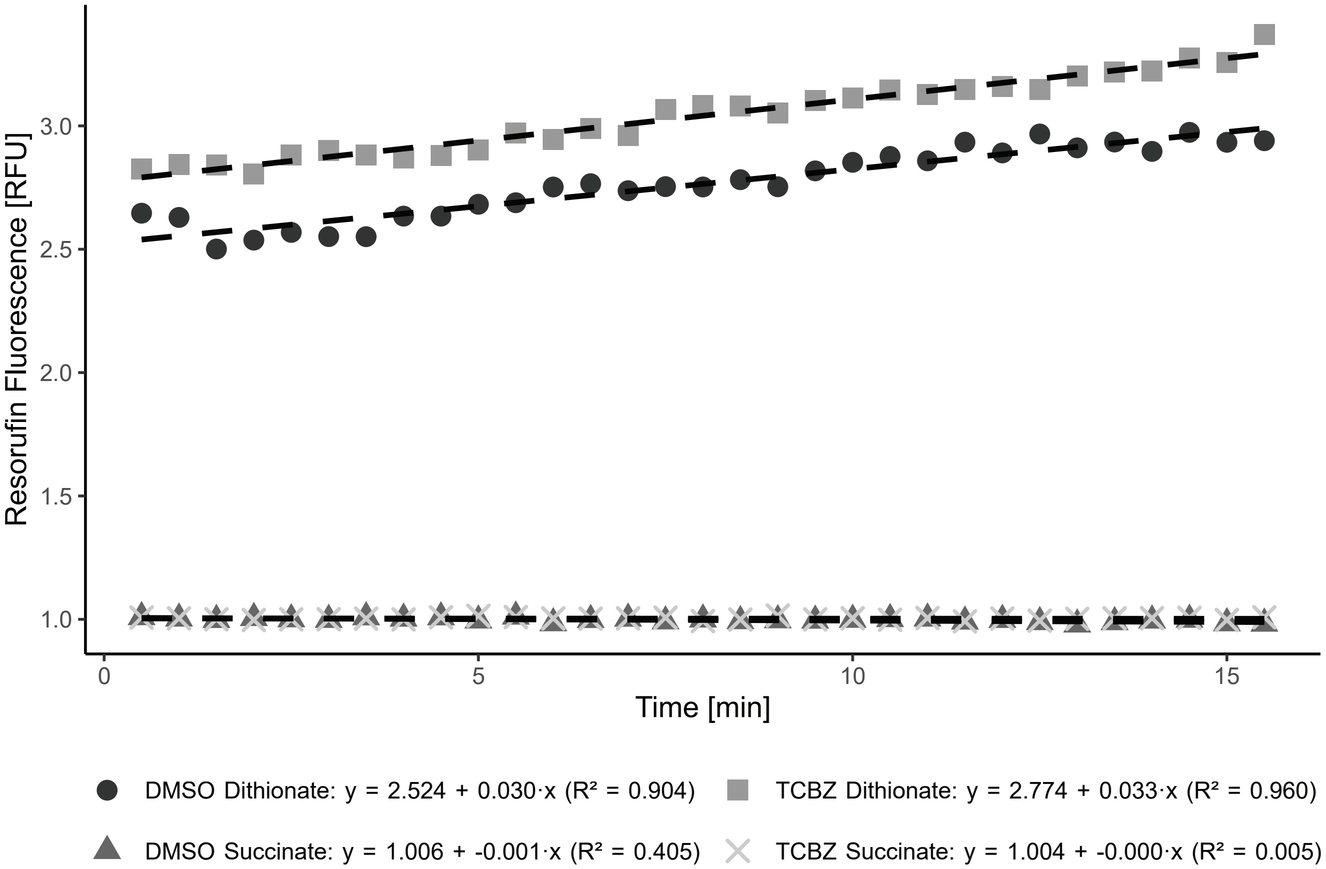

Fig. S1: Resazurin reduction confirmation assay showing that TCBZ does not inhibit resazurin reduction in the absence mitochondrial dehydrogenases.

		biological replicate 1	biological replicate 2	biological replicate 3	Bonferroni-adjusted *p*-values (compared to DMSO)
Echinococcus multilocularis	DMSO	100.0	100.0	100.0	
	Malonate	22.6	7.5	14.9	7.76E-04
	ABZ	94.1	103.1	89.4	6.24E+00
	ABZ-SO	92.9	95.1	83.8	1.02E+00
	$ABZ\text{-}SO_2$	93.5	93.3	88.0	1.76E-01
	BNM	92.3	89.8	91.5	4.97E-03
	CMB	88.1	83.5	78.5	7.19E-02
	CZM	84.1	93.0	92.3	4.54E-01
	FBT	87.9	87.9	82.5	2.95E-02
	FBZ	88.0	96.6	97.0	2.01E+00
	MBZ	83.5	89.1	82.7	3.39E-02
	NCZ	90.7	86.4	80.2	1.84E-01
	OBZ	84.3	95.9	68.8	1.83E+00
	OFZ	86.6	92.8	93.3	2.58E-01
	OMP	90.1	89.6	92.6	1.06E-02
	PBZ	86.2	90.7	92.7	1.22E-01
	TCBZ	15.1	18.4	44.2	2.46E-02
	TCBZ-SO	68.4	60.8	48.5	4.05E-02
	$TCBZ\text{-}SO_2$	43.3	41.7	54.0	2.96E-03
	THB	88.7	91.2	82.3	1.67E-01
	UBZ	85.9	99.4	88.4	1.96E+00
Fasciola hepatica	DMSO	100.0	100.0	100.0	
	Malonate	9.9	60.1	30.2	7.19E-02
	ABZ	113.7	100.6	101.4	1.99E+00
	ABZ-SO	119.4	96.6	99.6	3.55E+00
	$ABZ\text{-}SO_2$	119.3	98.2	101.0	2.83E+00
	TCBZ	1.3	5.7	2.2	1.53E-06
	TCBZ-SO	64.2	49.3	51.5	4.42E-03
	$TCBZ\text{-}SO_2$	53.3	43.5	42.9	6.60E-04
Toxocara cati	DMSO	100.0	100.0	100.0	
	Malonate	15.6	26.8	64.1	8.14E-02
	ABZ	103.6	92.5	104.7	6.63E+00
	ABZ-SO	101.3	88.3	95.7	1.83E+00
	$ABZ\text{-}SO_2$	98.8	100.4	97.7	1.73E+00
	TCBZ	37.8	60.7	58.4	1.97E-02
	TCBZ-SO	34.8	59.5	59.5	2.87E-02
	$TCBZ\text{-}SO_2$	32.3	58.2	54.7	2.18E-02
Rattus norvegicus	DMSO	100.0	100.0	100.0	
	Malonate	13.1	42.6	42.6	1.67E-02
	ABZ	86.3	127.5	83.7	6.70E+00
	ABZ-SO	103.1	116.9	88.0	5.34E+00
	$ABZ\text{-}SO_2$	113.4	101.0	94.9	4.17E+00
	TCBZ	42.7	47.2	47.5	2.63E-05
	TCBZ-SO	35.9	32.1	41.8	1.62E-04
	$TCBZ\text{-}SO_2$	26.6	33.3	20.5	2.70E-04
Mus musculus	DMSO	100.0	100.0	100.0	
	Malonate	6.4	-6.3	3.9	1.02E-04
	ABZ	105.9	84.5	97.2	3.78E+00
	ABZ-SO	106.3	92.6	96.3	5.03E+00
	$ABZ\text{-}SO_2$	103.8	84.4	95.9	2.79E+00
	TCBZ	61.0	32.9	45.3	1.92E-02
	TCBZ-SO	73.6	55.8	69.0	2.17E-02
	$TCBZ\text{-}SO_2$	51.5	28.9	38.8	5.33E-03

Fig. S2: Data of SDH inhibition assay and respective *p*-values.

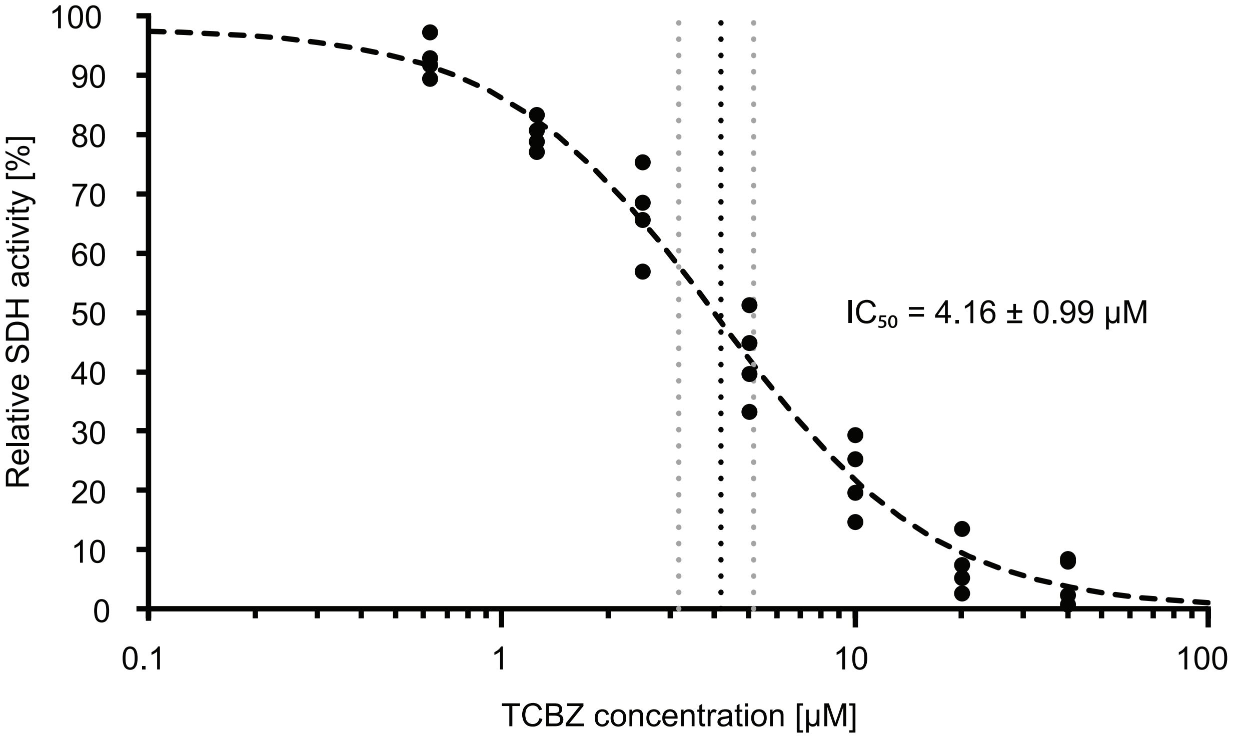

Fig. S3: SDH inhibition assays using enriched mitochondrial fractions of *E. multilocularis* metacestodes at various concentrations of TCBZ. Inhibitory concentrations were determined by fitting a four-parameter logistic. Data points are based on technical quadruplicates of a single biological replicate.

HsSDHA (*Homo sapiens*, P31040),
MmSDHA (*Mus musculus*, Q8K2B3),
RnSDHA (*Rattus norvegicus*, Q920L2),
EmSDHA (*Echinococcus multilocularis*, A0A068Y498),
FhSDHA (*Fasciola hepatica*, A0A4E0RX47),
TcSDHA (*Toxocara canis*, A0A0B2VZN8),
HsSDHB (*Homo sapiens*, P21912),
MmSDHB (*Mus musculus*, Q9CQA3),
RnSDHB (*Rattus norvegicus*, P21913),
EmSDHB (*Echinococcus multilocularis*, A0A068XXL3),
FhSDHB (*Fasciola hepatica*, A0A4E0R6D1),
TcSDHB (*Toxocara canis*, A0A183UKK7),
HsSDHC (*Homo sapiens*, Q99643),
MmSDHC (*Mus musculus*, Q9CZB0),
RnSDHC (*Rattus norvegicus*, Q641Z9),
EmSDHC (*Echinococcus multilocularis*, A0A068YIU3),
FhSDHC (*Fasciola hepatica*, A0A4E0RZS8),
TcSDHC (*Toxocara canis*, A0A183UD37),
HsSDHD (*Homo sapiens*, O14521),
MmSDHD (*Mus musculus*, Q9CXV1),
RnSDHD (*Rattus norvegicus*, Q6PCT8),
EmSDHD (*Echinococcus multilocularis*, A0A068YKN0),
FhSDHD (*Fasciola hepatica*, FhHiC23_g14243),
TcSDHD (*Toxocara canis*, A0A0B2USZ2).

Fig. S4: List of UniProt accession numbers of the sequences used for the sequence alignment.

SDHA

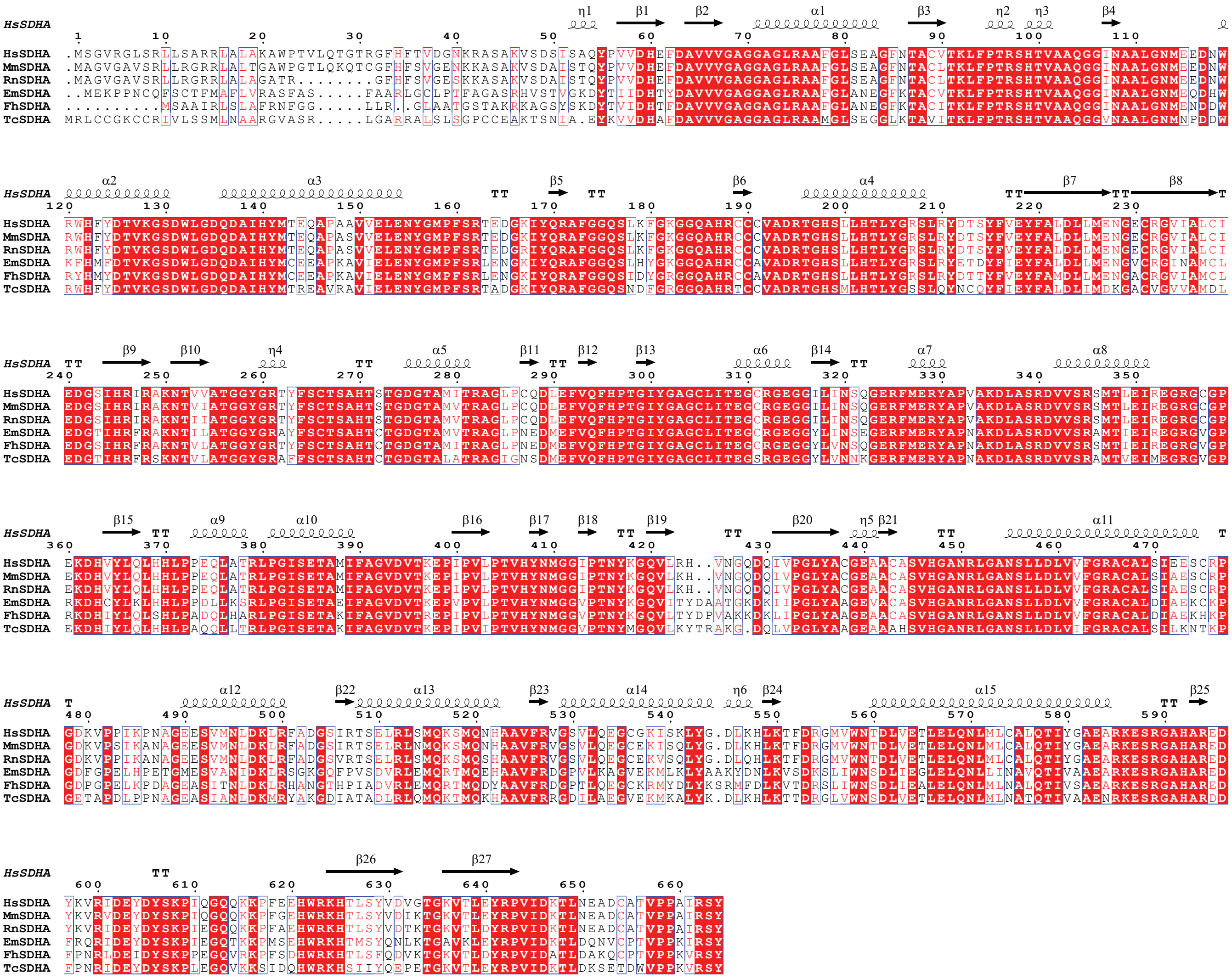

Fig. S5: Sequence alignments for subunit A (succinate dehydrogenase [quinone] flavoprotein subunit) of mitochondrial complex II.
